# Conservation of the links between gene transcription and chromosomal organization in the highly reduced genome of *Buchnera aphidicola*

**DOI:** 10.1186/1471-2164-8-143

**Published:** 2007-06-04

**Authors:** José Viñuelas, Federica Calevro, Didier Remond, Jacques Bernillon, Yvan Rahbé, Gérard Febvay, Jean-Michel Fayard, Hubert Charles

**Affiliations:** 1 UMR 203 Biologie Fonctionnelle Insectes et Interactions, IFR41, INRA, INSA-Lyon, F-69621 Villeurbanne, France; 2 Laboratoire de Dynamique des Machines et des Structures, INSA-Lyon, F-69621 Villeurbanne, France; 3 DTAMB, Université Claude Bernard Lyon-1, F-69622 Villeurbanne, France

## Abstract

**Background:**

Genomic studies on bacteria have clearly shown the existence of chromosomal organization as regards, for example, to gene localization, order and orientation. Moreover, transcriptomic analyses have demonstrated that, in free-living bacteria, gene transcription levels and chromosomal organization are mutually influenced. We have explored the possible conservation of relationships between mRNA abundances and chromosomal organization in the highly reduced genome of *Buchnera aphidicola*, the primary endosymbiont of the aphids, and a close relative to *Escherichia coli*.

**Results:**

Using an oligonucleotide-based microarray, we normalized the transcriptomic data by genomic DNA signals in order to have access to inter-gene comparison data. Our analysis showed that mRNA abundances, gene organization (operon) and gene essentiality are correlated in *Buchnera *(i.e., the most expressed genes are essential genes organized in operons) whereas no link between mRNA abundances and gene strand bias was found. The effect of *Buchnera *genome evolution on gene expression levels has also been analysed in order to assess the constraints imposed by the obligate symbiosis with aphids, underlining the importance of some gene sets for the survival of the two partners. Finally, our results show the existence of spatial periodic transcriptional patterns in the genome of *Buchnera*.

**Conclusion:**

Despite an important reduction in its genome size and an apparent decay of its capacity for regulating transcription, this work reveals a significant correlation between mRNA abundances and chromosomal organization of the aphid-symbiont *Buchnera*.

## Background

Past genomic studies have comprehensively described the organization of the bacterial chromosome, for example in terms of gene localization, order and orientation. The degree of organization has been shown to increase with genome size, overall GC composition and the presence of nucleoid-binding proteins [1]. This organization of the chromosome can be described as being an adaptive and functional tool, essential for the survival of the bacterial cell. More precisely, several studies have identified strand asymmetries in the distribution of genes between the leading and the lagging strand of DNA (for a review see Rocha [2]). Indeed, bacterial genomes carry, on average, from 78% (for genomes containing the polymerase PolC) to 58% (for the other genomes) of their genes on the leading strand [3]. This bias is even more important when the essentiality of genes is taken into account, and essential genes distribution bias reaches 76% and 94% in *Escherichia coli *and *Bacillus subtilis *respectively [4]. The asymmetry of the distribution of the genes between the two strands of DNA is explained as being a means of minimizing interruptions in gene transcription due to collisions between the DNA and RNA polymerases. The most generally accepted hypothesis is that co-directional collisions on the leading strand have a weaker effect on RNA polymerase processivity than the head-on collisions occurring on the lagging strand. This "replicational selection" should guarantee that genes on the leading strand, particularly the essential ones, are efficiently transcribed [5-8].

Gene organization in the bacterial chromosome has very well known effects on transcription and a standard example is the organization of genes into operons, which allows for a sophisticated regulation of gene expression [9]. It has also been shown that neighbouring genes in a bacterial chromosome tend to be co-expressed, even if they are not in the same operon [10], suggesting the existence of a "supra-operonic" organization [11]. This is a perfect illustration of the mutual influence between gene expression levels and chromosomal organization in bacteria. Another example is the significant effect of DNA supercoiling on transcription. Recent studies have suggested that the dynamical structure of the nucleoid acts as a "transcription factor" in *E. coli *[12], and is probably responsible for the presence of spatial transcriptional patterns in free-living bacteria [13-15].

While these observations were made on free-living bacteria, very few data exist on symbiotic bacteria characterized by reduced genomes, shaped by their adaptation to the host metabolic requirements and by their high evolution rate. *Buchnera aphidicola*, the endosymbiont of the aphids, is one of the best studied examples of an intracellular bacterium with a reduced genome [16,17]. The genomes of four *Buchnera *from different aphid species have been sequenced so far, with sizes ranging from 0.42 to 0.65 Mb [18-21]. In all the *Buchnera *genomes, most of the genes involved in the biosynthesis of essential amino acids (EAAs) that the insect cannot synthesize, or find in sufficient amounts in the phloem sap, were retained, whereas almost all genes regulating their expression were lost [22]. In *Buchnera *from the pea aphid *Acyrthosiphon pisum *(BAp), among the 608 chromosomal genes, 56% are located on the leading strand DNA. This observation reveals a small gene-strand bias, although equivalent to that of its closely related free-living bacteria:*E. coli *(55%) [23].

Gene essentiality has been defined by experimental techniques in cultivable bacteria, such as *E. coli *for which a large repertoire of null mutants is available. For a small number of genes, even in the absence of mutants, essentiality is defined on the basis of their function. For example, genes encoding ribosomal proteins are usually described as essential, whereas genes involved in the flagellar apparatus are classified as non-essential in bacteria [24]. Due to the specific lifestyle and symbiotic functions of *Buchnera*, the definition of essentials genes for this bacterium is particularly difficult. For this reason, we considered the minimal gene set for supporting bacterial life proposed by Gil *et al*. [25] as the most appropriate for our study. Indeed, the determination of this set is based on comparative analyses of five bacterial insect endosymbionts with small genomes (among them three *Buchnera *genomes), one of the smallest bacterial genome sequenced (*Mycoplasma genitalium*), and the essential gene lists available for *B. subtilis *and *E. coli *in the literature. Based on this minimal set of essential genes, BAp harbour 190 essential encoding-protein genes and 60% of them are located on the leading strand. This indicates a strand bias for essentiality, albeit lower than that observed in free-living bacteria. Recent transcriptomic analyses have shown that, despite a low response to different physiological conditions imposed on the aphid host, *Buchnera *retained the capability to express genes at different levels in basal conditions. This expression seems to be correlated to genome organization in the putative transcription units (pTU), even in the absence of many of the known transcriptional regulatory proteins [26].

This work focuses on studying the relationships between gene transcription and chromosome organization in the highly reduced genome of *Buchnera*, by coupling genomic information with transcriptomic data. For that, mRNA abundances were measured with a full-genome oligo-array dedicated to BAp. The basal transcriptome of *Buchnera *was then analysed regarding (i) the location of the genes on the leading/lagging strand, the gene essentiality and operon organization, (ii) the gene evolution rate (as measured by the GC rate), and (iii) the spatial location of genes along the chromosome. One crucial point in this study was the choice of the data normalization procedure. Since 2002, authors have described the possible advantages of genomic DNA (gDNA) standard for microarray data normalization in the investigation of microbial genomes [27-29]. This useful attractive standard is readily available, inexpensive, and invariant over time and from laboratory to laboratory. Moreover, the main advantage of the normalization by gDNA is that it takes into account the probe/target affinity in order to allow for inter-gene calibration and expression level comparisons. For these reasons, we have developed and validated in this study a "spot by spot" microarray data normalization method based on gDNA.

## Results

### Effectiveness of using genomic DNA for microarray data normalization

As no gene repeat occurs in the *Buchnera *genome [18], microarray hybridization with gDNA should produce equivalent fluorescent signals for all the *Buchnera *gene data set. Moreover, the fluorescence variability of gDNA hybridization should be low and mainly explained by the thermodynamic properties of the probes driving their target affinities. Indeed, supplemental Figure 1A of the Additional file 1 shows a shrunken fluorescent distribution for the slides hybridized with *Buchnera *gDNA, compared with the cDNA slides. gDNA fluorescence variability was analysed (ANOVA F-test and regression models) regarding the following parameters: probe GC rate, probe size, probe specificity, putative hairpin and homoduplex entropies (ΔG). We found that about 12% of gDNA signal variability could be significantly explained by the *Buchnera *probe GC rate: GC-rich probes are much more fluorescent than AT-rich ones (Additional file 1, supplemental Figure 1B). On the contrary, no significant influence on gDNA signals was found for the four other factors analysed (data not shown), indicating an optimal probe design with the software ROSO [30].

**Figure 1:**
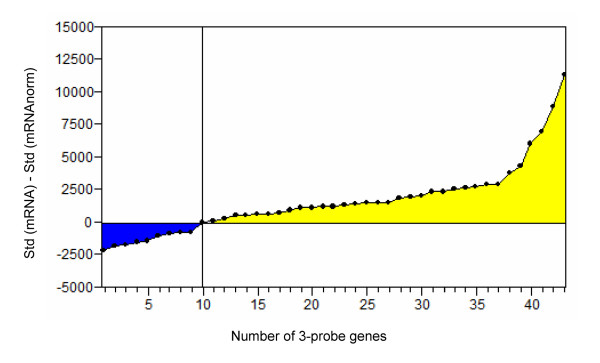
**Validation of the gDNA normalization procedure**. Differences (before and after gDNA normalization) of the standard deviations of the fluorescent signals for the 43 3-probe genes of the *Buchnera *oligo-array are reported here. Genes are ranged by increasing differences in standard deviation. The 34 genes of the yellow area (positive differences) show a reduction of their probe standard deviation after gDNA normalization.

The normalization procedure consists of weighting each spot with the inverse of the gDNA fluorescence signal. This is described in the method section. The normalization procedure does not affect the shape, the mean or the median of the mRNA fluorescent signal distribution (Additional file 2, supplemental Figure 2). We should mention that the difference in the expression levels between the least and the most highly expressed genes in *Buchnera *(more than 10 fold) confirmed the existence of a basal transcriptional regulation in the aphid endosymbiont.

**Figure 2:**
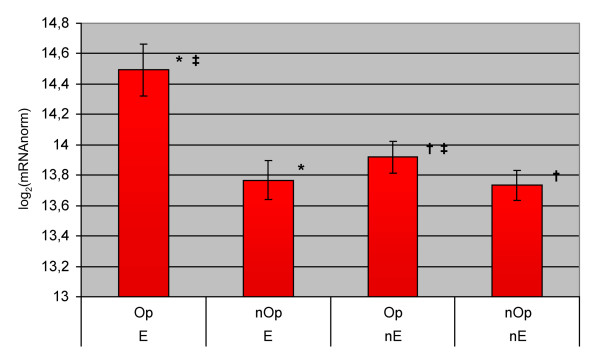
**Means of log_2 _nBGT levels in *Buchnera***. Genes are split into essential ("E") or non-essential ("nE") genes and predicted to be in putative operons ("Op") or not ("nOp"). The error bars indicate the 95% confidence interval of the mean. After applying the Bonferroni correction for multiple tests, the significant differences obtained by ANOVA and Likelihood ratio tests between two classes of genes are marked with the same symbol: * significant difference between "Op" and "nOp" for "E"; † significant difference between "Op" and "nOp" for "nE"; ‡ significant difference between "E" and "nE" for "Op"; no significant difference was observed between "E" and "nE" for "nOp".

Finally, we took advantage of the design of several probes (2 or 3) per gene in our microarray to validate the gDNA normalization procedure. Hence, we compared the within-gene fluorescence signal standard deviations of all the 3-probe genes, before and after normalization. The results presented in Figure 1 show, after normalization, an overall reduction of the fluorescent signals variability for about 79% of the genes. Close results were obtained for the 2-probe genes (65%, data not shown). Although low mRNA abundances seemed to be the major source of failure for gDNA normalization (data not shown) no intensity correction was included because the estimation of variability was not enough powerful with only 2 or 3 different probes per gene.

### Analysis of the links between *Buchnera* normalized mRNA abundances and chromosome organization

#### DNA strand position, putative operons and gene essentiality

In this part of the work, we analysed the effect of gene DNA strand position, operon organization and gene essentiality on the variability of the basal mRNA abundances in *Buchnera*.

Analysis of the pTU factor alone revealed that normalized basal gene transcription (nBGT) variation was always smaller within pTU than between them, indicating that genes belonging to the same pTU tend to be transcribed at similar rates (ANOVA F-test, R^2 ^= 0.640, *P*-value < 10^-4^). Analysis of essentiality alone revealed that the *Buchnera *genome contains 32% of essential genes versus only 6% for the *E. coli *genome [31].

Using a complete ANOVA model (including essentiality, strand position and pTU factors), we have shown that pTU and essentiality are significant factors, whereas no significant effect was observed for strand position (Table 1). Gene distributions in the different categories are presented in the supplemental Figure 3 of the Additional file 3. The analysis was then split at each level of each significant factor (operon/singleton, and essential/non-essential). The data were analysed by fluorescence comparison (Figure 2) and by distribution comparison (data not shown) with similar conclusions. When we tested the factor "pTU" on *Buchnera *mRNA abundances we found that the genes in pTU were more highly expressed than genes in singletons for both essential and non essential genes. Analysing the "essentiality" factor, we showed that, for genes in pTU, the highest expressed ones are essential, whereas this effect was not true for singletons (Figure 2).

**Table 1:** Effects and interactions of the global ANOVA model fitted on the *Buchnera* log_2 _nBGT levels

**Source**	**DF **^†^	**Sum of Squares **^‡^	**F Ratio **^∥^	***P*-value**
Operon	1	22.216	43.204	< 0.0001 *
Essentiality	1	9.627	18.722	< 0.0001 *
Strand	1	0.009	0.017	0.897
Essentiality × Operon	1	7.246	14.092	0.0002 *
Essentiality × Strand	1	0.055	0.107	0.744
Strand × Operon	1	0.280	0.545	0.461
Essentiality × Strand × Operon	1	0.653	1.271	0.260
Residual error	533	274.067		

*: significant *P*-value for 95% confidence limits, †: degrees of freedom for each source of variation, ‡: sum of squared distances for each source of variation, ∥: model mean square divided by the error mean square

**Figure 3:**
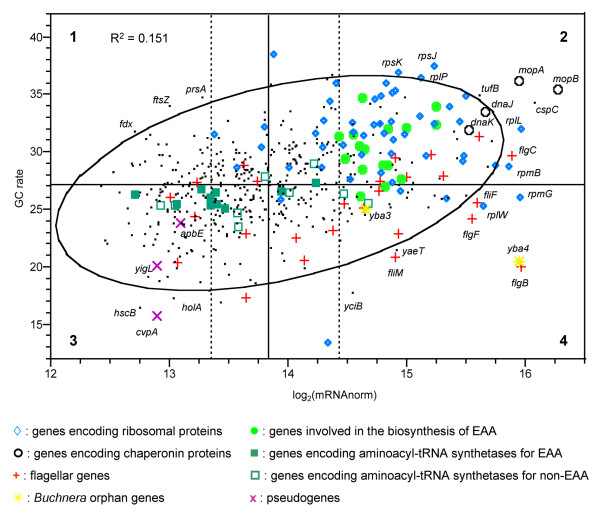
**Plot of log_2 _nBGT levels versus GC rate for *Buchnera *genes**. The bivariate normal ellipse (% = 0.95) is drawn in black and the four main areas are delimited by the median of the gene GC rate parameter and by the first and fourth quartile of the mRNA abundance level variable. Pearson correlation coefficient (R^2^) is shown. The names of the genes outside the ellipse in the four zones are specified.

#### Gene evolution rate

In the second part of this work we have analysed the relationship between nBGT levels and gene evolution rates in *Buchnera*. Gene evolution rates were estimated here by the GC content of genes instead of by non-synonymous substitution rates (Ka), since both parameters are highly correlated in *Buchnera*. Figure 3 shows that mRNA abundances and GC content are correlated (R^2 ^= 0.151, *P*-value < 10^-4^): highly expressed genes are the most GC rich (the most highly conserved in *Buchnera*). More precisely, areas 1 and 2 in Figure 3 correspond to genes that are slowly evolving and that are weakly or highly expressed respectively, whereas areas 3 and 4 correspond to genes that are rapidly evolving and that are weakly or highly expressed respectively. In the second area we found almost all genes encoding ribosomal proteins (among them: *rplL*, *rplP*, *rpmB*, *rpsJ*, *rpsK*) but also genes encoding chaperone proteins (*dnaK*, *dnaJ *and *mopA*,*mopB*). An important proportion of the flagellar genes seem to evolve rapidly: 16 out of 26 in *Buchnera *(among them 9/12 *fli *genes) and almost all of them are highly expressed (11 out of 16). Moreover, 11 out of these 16 highly evolving flagellar genes are located on the leading strand (among them 8 *fli *genes). Interestingly, the only two *Buchnera *orphan genes (*yba3 *and *yba4*), are highly expressed and show a low GC content (area 4). The genes encoding the enzymes involved in the biosynthesis of EAAs generally evolve slowly and show high mRNA abundances. Among them, we have observed that the genes belonging to the two operons involved in the biosynthesis of isoleucine and valine are particularly highly expressed and well conserved in *Buchnera *(area 2). Moreover, *ileS*, *valS*, and six other genes encoding aminoacyl-tRNA synthetase for EAAs are weakly expressed, whereas genes encoding aminoacyl-tRNA synthetase for non-EAAs are moderately or highly expressed (Median test, *P*-value = 1.4 × 10^-2^). Finally, area 3 includes pseudogenes, such as *apbE*, *cvpA*, and *yigL*.

#### Spatial location of genes along the chromosome

**Figure 4:**
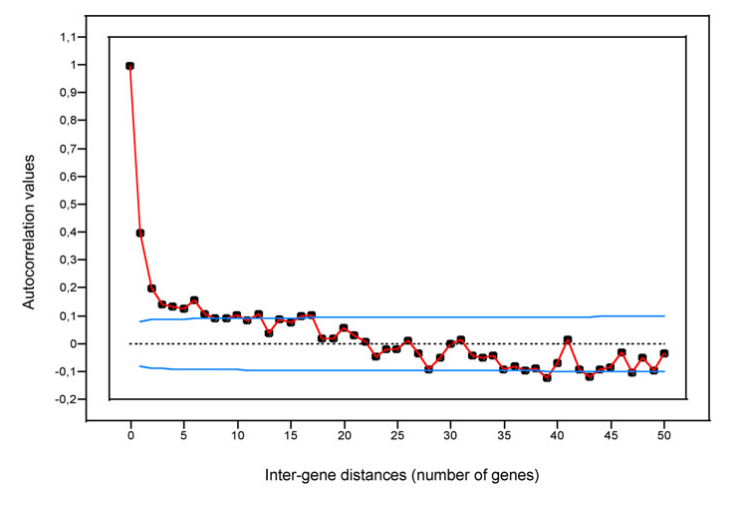
**Autocorrelation function of the spatial series corresponding to the *Buchnera *log_2 _nBGT along the chromosome**. The blue lines represent ± 2 standard errors for approximate 95% confidence limits (i.e., values above the threshold are significant).

The question we addressed in the final part of this work was the existence of spatial periodic patterns of transcriptional activity along the chromosome of *Buchnera*. As has been reported for the genomes of free-living bacteria [15], spectral analysis revealed significant periodic components in the gene transcription levels along the chromosome of the aphid endosymbiont (Fisher's Kappa test, *P*-value = 4.6 × 10^-4^; Bartlett's Kolmogorov-Smirnov test, *P*-value < 10^-2^). To substantiate these results, we analysed the autocorrelation function of nBGT levels with regard to gene order along the chromosome on a smaller scale (with inter-gene distances ranging from 1 to 50 genes). Figure 4 shows that closely spaced genes on the chromosome, and more precisely groups of 2 to 8 genes, have highly correlated transcriptional patterns along the chromosome of *Buchnera*. We also observed that this correlation decreases with the size of the groups. Such small autocorrelated structures (of about 2–8 genes) might be interpreted as being an operon effect in *Buchnera*, as it has been reported for *E. coli *[15].

**Figure 5:**
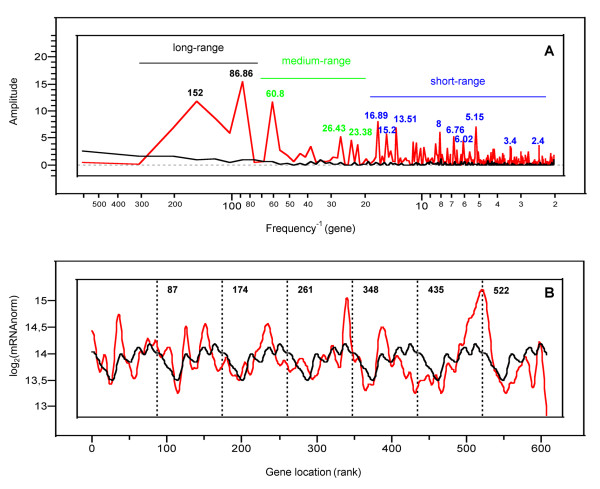
**Spatial periodic transcriptional patterns in the genome of *Buchnera***. (A) Periodogram of *Buchnera *log_2 _nBGT. The main periods composing long, medium and short-range spatial patterns are respectively specified in black, green and blue. For comparison, the periodogram of log_2 _gDNA signals is shown in black. (B) Averaged smoothed signal related to the period "86.86" is drawn in black, along the chromosome, whereas *Buchnera *transcript abundances (real data) appear in red.

More generally, the plot of the spectral density of the autocorrelation function displays periods contained between 2 and 152 gene lengths. These periods were grouped as long-range, medium-range and short-range periods on Figure 5A. To illustrate these results, Figure 5B presents the averaged and smoothed signal of the seven 87 gene segments constituting the period "86.86" on the complete *Buchnera *chromosome. On this figure, the black curve corresponding to the period "86.86" shows periodic oscillations with minima at a distance of 29, 116, 203, 290, 377, 464 and 551 genes. These minima, due to a specific location of the genes along the chromosome, are superimposed with those of the red curve which corresponds to the experimental *Buchnera *transcript abundances. The red curve also shows several maxima, absent in the black one, belonging to particular functional categories such as genes encoding ribosomal proteins and flagella.

Some recent papers [32-34] reported that spurious periodicities in transcriptomic data can be attributed to the spatial arrangements of the probes on an oligo-array. Even if by construction our gDNA normalization procedure is appropriate to take into account this bias, we checked the possible presence of periodic components in gDNA signals. The absence of significant periodicities for gDNA (black curve of Figure 5A) supports the idea that the observed spatial patterns for mRNA signals are proper to the *Buchnera *transcriptome.

**Figure 6:**
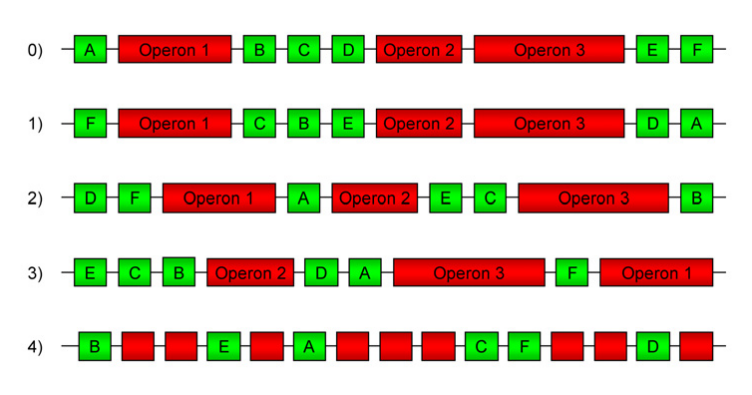
**Different kinds of simulated permutations of gene positions applied to the *Buchnera *chromosome**. Singletons are drawn in green whereas genes predicted to be in operons are drawn in red. 0: Original location of genes on the chromosome, 1: permutation preserving both rank and the spacing of the pTU to each other but modifying the rank of the genes between the pTU, 2: permutation preserving the rank but not the spacing between the pTU, 3: permutation of both the rank and the location of pTU, 4: gene positions on the chromosome randomly assigned.

To ascertain whether the observed periodic patterns were only caused by the pTU in *Buchnera*, we performed different simulated permutations of the *Buchnera *gene positions (Figure 6) and we ran autocorrelation and spectral analyses on the resulting data. The first permutation preserved both the rank and the spacing between the pTU and we only modified the location of the singletons; the second one conserved only the rank of the pTU and we modified the number of singletons between them; the third permutation changed both the rank and the location of the pTU; and, finally, in the fourth one all the genes were randomly distributed. From the real series to the third permutation ("Perm. 0" to "Perm. 3"), we observed a decrease, from 8 to 2, in the maximum size of the autocorrelated groups of genes (Table 2). Interestingly, the mean size of the pTU in *Buchnera *is around 2 (45 out of the 82 pTU are doublets). Moreover, analysing the different spectral density (periodograms) it appeared that the presence of spatial periodic patterns of transcriptional activity in *Buchnera *became less and less pronounced, and eventually disappeared, when the genes were randomly assigned along the *Buchnera *chromosome (Table 3, and supplemental Figure 4 of the Additional file 4). The periods which are preferentially affected by the permutations are: (1) the short-range periods for the first permutation (shuffling singletons but preserving the operon backbone), (2) the medium-range periods for the second one (slight degradation of the operon backbone), and (3) the long-range periods for the third permutation (operons are shuffled). This observation suggests that, even if the structure in putative operons seems to play a key role in the existence of transcriptomic spatial patterns in *Buchnera*, the order and the spacing of singletons around these pTU are also important in establishing the observed periodicities. Moreover, genes located in the neighbourhood of operons seems to be co-expressed with the operons in kinds of "supra-operonic" structures as it was previously suggested by the work of Moran *et al*. [35].

**Table 2:** Autocorrelation values of *Buchnera *nBGT levels for the different permutations of gene positions

**Inter-gene distances**	**Perm. 0**	**Perm. 1**	**Perm. 2**	**Perm. 3**	**Perm. 4**
1	0.3992 *	0.2842 *	0.2498 *	0.2240 *	-0.0249
2	0.1977 *	0.1474 *	0.1385 *	0.1386 *	0.0467
3	0.1430 *	0.1346 *	0.0834	0.0582	0.0311
4	0.1336 *	0.0987 *	0.0993 *	0.0067	0.0078
5	0.1277 *	0.1165 *	0.1571 *	0.0185	0.0595
6	0.1577 *	0.1153 *	0.0968 *	-0.0069	-0.0314
7	0.1079 *	0.0983 *	0.0375	0.0745	-0.0070
8	0.0925 *	0.0093	0.0279	0.0415	-0.0934 *
9	0.0920	0.0259	0.0345	0.0237	-0.0013
10	0.1025 *	0.0784	0.0387	-0.0063	-0.0502
11	0.0866	0.0081	0.0121	0.0266	-0.0291
12	0.1071 *	0.0598	0.0854	-0.0336	0.0014
13	0.0372	0.0428	0.0632	-0.0822	0.0508
14	0.0888	0.0689	0.0554	-0.0703	0.0079
15	0.0786	0.0891 *	0.0079	-0.0469	-0.0382

0: Actual data; 1: conserved operons, order and spacing; 2: conserved operon order; 3: conserved operons; 4: gene scrambling (see text and Figure 6 for details). Autocorrelation values are reported here at operon size scale (1 to 15 inter-gene distances).

*: significant *P*-value for 95% confidence limits

**Table 3:** Results of the spectral analysis testing the periodicity of *Buchnera *log_2 _nBGT

	**Fisher's Kappa**	**Bartlett's Kolmogorov-Smirnov**
Perm. 0	13.148 **	0.275 **
Perm. 1	10.930 **	0.194 **
Perm. 2	9.174 *	0.177 **
Perm. 3	6.941	0.173 **
Perm. 4	6.595	0.041

The periodicity was tested for the original location of genes on the chromosome or according to the different simulated permutations of gene positions. 0: Actual data; 1: conserved operons, order and spacing; 2: conserved operon order; 3: conserved operons; 4: gene scrambling (see text and Figure 6 for details).

**: significant *P*-value for 99% confidence limits, *: significant *P*-value for 95% confidence limits

## Discussion

To our knowledge, we have presented here the first analysis showing i) a representation of whole-genome transcriptional data in *Buchnera *and ii) the links between mRNA abundances and chromosomal organization in a highly reduced bacterial genome, on the basis of experimental data. The large number of prokaryotic genome sequences available in databases has made it possible to study these links in many bacterial chromosomes. Using codon adaptation index (CAI) values computed from ribosomal proteins, Rocha and Danchin have shown that, in *B. subtilis *and in *E. coli*, the importance of the expressiveness in determining the localization of the genes on the leading strand is negligible, or even absent, when essentiality is taken into account [36]. They have confirmed these results for other sequenced genomes, with the exception of some non-free-living bacteria among which *Buchnera *was one of the most important [4]. However, the authors underlined the difficulty of obtaining correct CAI values in the genomes of intracellular bacteria, which generally do not show sufficient codon usage bias. Moreover, another explanation of the exceptions they found for *Buchnera *can be attributed to authors' assignment of gene essentiality, which was exclusively based on the homology with *E. coli*. Indeed, while Rocha *et al*. [4] found no essential genes strand distribution bias in *Buchnera*, we found a bias of 60% using the minimal gene set proposed by Gil *et al*. [25]. More recently, Price *et al*. [37] have revisited the question of gene-strand bias in bacteria, using gene expression microarray data, and they have shown that, in *B. subtilis *and *E. coli*, the genes in operons on the leading strand DNA are more highly expressed than genes in operons on the lagging strand. This observation was true for both essential and non-essential genes.

In *Buchnera*, we found that highly expressed genes are generally the essential genes within pTU. Independently of the essentiality factor, the genes within pTU are more highly expressed than singletons. We also found that for genes within pTU the essential genes are more highly expressed than non essential ones whereas this effect was not observed for singletons. These results underline the conservation of a coherent relationship between mRNA abundances and gene essential functions in the reduced genome of *Buchnera*.

In this work we also analysed the relationship between mRNA abundances and genes GC ratio in *Buchnera *(taken as an indicator of gene evolution rate). By combining transcription analysis, evolution rates and comparative genomics, we were able to define new candidates for the essential gene set of *Buchnera*. In bacteria, genes encoding ribosomal, cell division and chaperone/protease proteins are considered as essential and they are also known to be constitutively highly expressed. As we expected, our data showed that almost all genes encoding ribosomal proteins, and genes encoding chaperonins, are relatively well conserved and also highly expressed in *Buchnera*. Interestingly, among the most highly conserved and expressed genes, we found genes involved in the biosynthesis of EAAs and, in particular, all the genes of the isoleucine and valine pathway. Also remarkable is that the genes encoding the aminoacyl-tRNA synthetases for 8 out of the 10 aphid EAAs are weakly expressed, whereas genes encoding aminoacyl-tRNA synthetases for non-EAAs are either moderately or highly expressed. A possible explanation for this observation is that *Buchnera*, which is a relatively slow-growing bacterium, does not necessitate high rates of protein production and constitutively synthesizes EAAs in order to furnish them to the aphid. Reducing the abundance of specific aminoacyl-tRNA synthetases might increase the concentration of free EAAs in *Buchnera *cells facilitating the transport of these amino acids to the aphid host cell. This observation is reminiscent of a similar putative adaptive response of *Buchnera*, which selectively underexpresses *pheT *under aromatic EEAs shortage [26].

In our study, we also found that the orphan genes *yba3 *and *yba4 *seem to evolve rapidly and are highly expressed, *yba4 *being one of the ten highest expressed genes in *Buchnera*. The conservation of these genes in BAp and in the *Buchnera *harboured by the aphid *Schizaphis graminum *(BSg), coupled with their high expression level, suggest that they could be of particular relevance in the symbiosis of *Buchnera *with the aphids *S. graminum *and *A. pisum*, as it has also been proposed by Shimomura *et al*. [38] for *A. pisum*.

Among the genes rapidly evolving (this work, Tamas *et al*. [19] and Reymond *et al*. [26]) and highly expressed in *Buchnera*, we found most of the flagellar genes. These data, taken together with recent experimental evidence that the *Buchnera *incomplete flagellar apparatus can function as a "protein transporter" [39], support the idea that flagellar genes are taking on new important functions in the symbiotic context. The flagellar gene set of BAp is composed of 26 genes of which *fliEFGHIJKMNPQR *and *flhAB *genes are located on the leading strand (except *fliE*), and *flgNABCDEFGHIJK *genes are located on the lagging strand DNA (except *flgN *and *flgA*). These flagellar genes are also conserved in BSg. However, *Buchnera *from a distant aphid lineage, *Baizongia pistaciae *(BBp), has lost five flagellar genes (*flgA*, *flgD*, *flgE*, *flgK*, *flgN*) but not any of the *fli *ones. Finally, *Buchnera *with the most dramatically reduced genome, from the aphid *Cinara cedri *(BCc), has lost the same genes as BBp but also four other *flg *genes (*flgB*,*flgC*, *flgG*, *flgJ*) and four *fli *genes (*fliE*, *fliJ*, *fliK*, *fliM*), hence preserving only a minimal type III virulence secretion system [21]. The evolutionary selection of the majority of the *fli *genes also suggests the possible importance of the new putative transport function of these genes in *Buchnera*. It is to note, that the most conserved *fli *genes among the *Buchnera *lineages, probably involved in the new function, are located on the leading strand, whereas most of the gene losses occurred on the lagging strand. Previous comparative genomics analyses [40,41] had tempted to dissect the evolutionary forces driving the genome organization in several *Buchnera *lineages (i.e., gene strand bias, gene and protein composition, gene expressivity, gene evolution rate and gene loss). Our results are partly consistent with these previous analyses as we found (1) highly conserved genes are highly expressed, (2) essential genes in pTU are highly expressed and probably preserved from mutations by purifying selection, and (3) positive selection may shape new "symbiotic" functions for some genes highly expressed and highly evolving. However, we reject the former idea, based on CAI analyses, that expressiveness is a factor driving gene strand bias in *Buchnera*.

Finally, an important result of this study was the discovery of spatial patterns of transcriptional activity in the chromosome of *Buchnera*: i.e., the transcription of the genes along the chromosome is determined according to spatial constraints. From autocorrelation and spectral analysis, four groups of spatial patterns can be defined: (i) autocorrelated short-range (between 2 and 8 genes), (ii) periodic short-range (up to 17 genes), (iii) periodic medium-range (between 23 and 61 genes) and (iv) periodic long-range (over 87 genes) structural components.

Autocorrelated short-range patterns, determined by the autocorrelation function of gene transcription levels, showed that genes spaced by a gene-to-gene distance of less than 8 have highly correlated expressions. As has been suggested for *E. coli *and *B. subtilis*, we propose for *Buchnera *that these correlations reflect the co-ordinated transcription of genes within operons [14,15]. This observation reinforces the result mentioned above concerning the conservation of functional transcription units in *Buchnera*. However, by permuting gene position along the chromosome, we observed that the organization of genes into putative operons is not sufficient to fully explain the observed periodic spatial patterns of transcription. Indeed, if the presence of spatial periodic components in *Buchnera *gene expression was only due to the conservation of operon structures, the modification of the order and/or the number of the singletons located between the pTU should not affect the periodicities and the autocorrelation values. We have shown, however, that these modifications reduced the spatial patterns, alleviating the importance of a high-order arrangement of all the genes along the chromosome of the endosymbiont.

Jeong *et al*. [14] have classified the transcriptional periods that they have found in *E. coli *into three categories: short-range (up to 16 genes); medium-range (100–125 genes); and long-range (600–800 genes). The existence of short periods, up to 17 genes in *Buchnera*, allows us to corroborate the hypothesis, proposed in a previous study on *B. subtilis *and on *E. coli*, that this short range element could be a property of the structural nucleoid common to other bacteria, corresponding to large DNA spirals on the nucleoid surface [15]. The medium and the long-range periods are shorter in *Buchnera *than those identified for free-living bacteria, which is probably due to the greatly reduced size of its genome. However, these two kinds of periods are not yet understood and do not correspond to the domains identified so far in the nucleoid [15]. The effect of the second and third simulated gene permutations (Perm. 2 and Perm. 3 on Figure 6) on the medium and long-range periods respectively, could be explained by the importance of the spatial location of the operons along the chromosome and by the neighbourhood of singletons that form "supra-operonic" structures in *Buchnera*. Moreover, the decrease of the maximum size of the autocorrelated groups of genes (Table 2), for the different permutations of gene positions, corroborates the hypothesis of "supra-operonic" structures in *Buchnera*. But this speculation needs to be more studied and experimentally confirmed. Finally, the observation of transcriptional periodic patterns, coupled with the conservation in its genome of some Nucleoid Associated Proteins (NAP) such as H-NS, IHF and Fis, suggest that *Buchnera *has maintained a nucleoid structure responsible for the differences in gene transcription levels in basal conditions. These three NAPs were previously found to be differentially expressed in *Buchnera *[26] facing nutritional constraints. However, their role in transcriptional regulation remains presently speculative in the aphid endosymbiont.

## Conclusion

In conclusion, the analysis of mRNA abundances with regard to chromosomal organization in *Buchnera *has shown, despite an important reduction in its genome size and an apparent decay of its transcriptional regulatory capacity, a conservation of the relationship between these two parameters in the aphid symbiont. Our work shows that the organization of the genes into operons and their essential functions influence transcription in *Buchnera*, whereas no significant leading/lagging strand bias was observed. This work also underlines the difficulties in defining gene essentiality in intracellular symbiotic bacteria and the importance of an additional specific gene set for BAp. Finally, we showed the existence of a transcriptional periodicity along the chromosome of *Buchnera*. From these results, comparative analyses performed on *Buchnera *taken from other species of aphids, but also on other endosymbiotic bacteria, such as *Blochmannia *spp. from the carpenter ant or *Wigglesworthia *spp. from the tsetse fly would establish whether the results presented here are a common trait of the insect endosymbionts.

## Methods

### Aphid rearing

A long-established parthenogenetic clone (LL01) of *A. pisum *was maintained at 21°C, with a 16 hour light photoperiod, on *Vicia fabae*. It was shown to be free of any of the five taxa of secondary endosymbionts identified to date. For these experiments, we used aphids which were reared from birth to day-7 on the plant (basal conditions of gene expression in *Buchnera*) and *Buchnera *were purified from their host as described by Charles and Ishikawa [16].

### Microarray experiments

#### Nucleic acids purification and labelling

The protocols for RNA extraction and labelling have been previously described by Calevro *et al*. [42]. Briefly, total RNA was purified using the Trizol method and any possible gDNA contaminants were removed using DNase RQ1 RNase-free (Promega, Madison, WI, USA). Total RNA was subsequently purified on an RNeasy^® ^column (Qiagen, Hilden, Germany). Then, 15 μg of total RNA were indirectly labelled by incorporating aminoallyl-dUTP into reverse transcript cDNA (Amersham Biosciences, Piscataway, NJ, USA). Finally, Cy3 fluorescent dyes (Amersham) were coupled to the targets prior to purification on an Autoseq™ G-50 column (Amersham).

Genomic DNA was treated with RNase A (Sigma-Aldrich, St Quentin Fallavier, France) and classically purified with phenol/chloroform/isoamyl alcohol (25:24:1, v/v/v) (Q-Biogene, Irvine, CA, USA). After testing different concentrations of gDNA (from 1 to 6 μg), a concentration of 5 μg was chosen for the normalization of mRNA signals (data not shown). gDNA labelling was performed using the Nick Translation Kit (Amersham) and by directly incorporating dUTP-Cy3 (Amersham). Non-incorporated fluorescent dyes were eliminated by purification on an Autoseq™ G-50 column.

#### Slide preparation and hybridization

The *Buchnera *oligo-array is composed of 6144 spots, including two probes per *Buchnera *gene that were designed by ROSO software [30]. A third additional probe was designed for specific genes chosen for their function in the symbiotic relationships (amino acid biosynthetic genes and putative transporters). The 35-mer oligonucleotide probes were printed, as quadruplets, by two different pins onto Quantifoil Micro Tools aldehyde slides (Interchim, Montluçon, France). Positive and negative controls were spread all over the slide.

The slides were hybridized using an automated Ventana Discovery station (Ventana Medical Systems, Illkirch, France) at 45°C for 8 hours. Following hybridization, the slides were washed with solutions of variable stringency, dried by centrifugation and scanned for fluorescence using a GeneTac LSIV scanner (Genomic Solutions, Huntingdon, UK). The signal intensity value for each spot (pixel medians) was recorded and qualitatively analysed with GenePix Pro 4.1 software (Axon Instruments, Foster City, CA, USA).

Four slides were hybridized: 2 with *Buchnera *gDNA and 2 with cDNA. Microarray data are available in the Array Express database, accession E-TABM-193.

### Data analysis

#### Genomic DNA normalization

Fluorescence data were first filtrated and spots of low quality were removed. In this study we did not consider plasmidic genes which are present in several copies in the *Buchnera *genome, and we excluded from our data set genes encoding RNA genes (tRNAs, ribosomal RNAs and enzyme RNA components), due to their specific hybridization properties [43]. Moreover, 30 genes without a high quality signal were excluded. To be sure that these deletions do not introduce biases in our study, we checked that these genes were uniformly distributed within the different categories analysed (pTU, essentiality, strand, mRNA abundances). Data from the different repeated slides were then scaled and averaged. Fluorescence means were always equal for each print-tip group of the slides (no print-tip effect). For data normalization, we first calculated the mean of the gDNA fluorescent signals (*M*) to determine a weighting coefficient *K*_*i *_for the *S *spots (*i*) of the oligo-array. Finally, these weights were applied to *Buchnera mRNA*_*i *_signals in order to normalize them (*mRNAnorm*_*i*_). The following formulas were used to normalize the mRNA signals:

M=1S∑i=1SgDNAiKi=gDNAiMmRNAnormi=mRNAiKi
 MathType@MTEF@5@5@+=feaafiart1ev1aaatCvAUfKttLearuWrP9MDH5MBPbIqV92AaeXatLxBI9gBaebbnrfifHhDYfgasaacH8akY=wiFfYdH8Gipec8Eeeu0xXdbba9frFj0=OqFfea0dXdd9vqai=hGuQ8kuc9pgc9s8qqaq=dirpe0xb9q8qiLsFr0=vr0=vr0dc8meaabaqaciaacaGaaeqabaqabeGadaaakeaafaqabeqadaaabaGaemyta0Kaeyypa0ZaaSaaaeaacqaIXaqmaeaacqWGtbWuaaWaaabCaeaacqWGNbWzcqWGebarcqWGobGtcqWGbbqqdaWgaaWcbaGaemyAaKgabeaaaeaacqWGPbqAcqGH9aqpcqaIXaqmaeaacqWGtbWua0GaeyyeIuoaaOqaaiabdUealnaaBaaaleaacqWGPbqAaeqaaOGaeyypa0ZaaSaaaeaacqWGNbWzcqWGebarcqWGobGtcqWGbbqqdaWgaaWcbaGaemyAaKgabeaaaOqaaiabd2eanbaaaeaacqWGTbqBcqWGsbGucqWGobGtcqWGbbqqcqWGUbGBcqWGVbWBcqWGYbGCcqWGTbqBdaWgaaWcbaGaemyAaKgabeaakiabg2da9maalaaabaGaemyBa0MaemOuaiLaemOta4Kaemyqae0aaSbaaSqaaiabdMgaPbqabaaakeaacqWGlbWsdaWgaaWcbaGaemyAaKgabeaaaaaaaaaa@5EFF@

For the validation of the normalization procedure, we used two specific data sets of 43 and 371 genes with 3 and 2 different probes per gene respectively. These probes sets were characterized by high quality signals in the two types of hybridization (2 slides with gDNA and 2 slides with cDNA)

#### *Buchnera* genomic data

Sequence data for the *Buchnera aphidicola *genome, and the corresponding annotations, were retrieved from GenBank [44]. The definition of essential genes was based on the minimal gene set for supporting bacterial life (see introduction part) described in Gil *et al*. [25]. Putative operons in *Buchnera *have been manually identified by comparison with the *E. coli *genome. For that, each *E. coli *orthologous gene in *Buchnera *was searched for in EcoCyc database [45], and groups of *Buchnera *genes corresponding to transcription units in *E. coli *were collected. After removing the singletons, we determined 82 pTUs in *Buchnera *consisting of 2 to 13 genes. In this study, we estimated gene evolution rates from the GC content of genes instead of from the non-synonymous substitution rates (Ka) as the two parameters are highly correlated in the AT-rich genome of *Buchnera*, and because the GC content allows for estimations of the two orphan genes of *Buchnera*.

#### Spectral analysis and estimation of the periodicities

This analysis was performed with the platform "Time series" of the JMP 5.0.1.2 software (SAS Institute, Inc.). The presence of spatial periodic components in *Buchnera *gene expression levels was tested by Fisher's Kappa and Bartlett's Kolmogorov-Smirnov statistical tests. Fisher's Kappa tests the null hypothesis that the series is Gaussian white noise against the alternative hypothesis that the series contains an added deterministic periodic component of unspecified frequency. The Bartlett's Kolmogorov-Smirnov test compares the normalized cumulative periodogram with the cumulative distribution function of the uniform (0, 1) to test the null hypothesis that the series is white noise. These tests were also applied to the *Buchnera *data set following different permutations of the gene positions, preserving or not the rank and the spacing between the pTU along the chromosome. To determine spatial patterns, we calculated the autocorrelation function of the *Buchnera *transcriptional spatial series. The autocorrelation for the *k*^th ^lag (inter-gene distance) is given by:

rk=ckc0whereck=1N−k∑x=k+1N(yx−y¯)(yx−k−y¯)
 MathType@MTEF@5@5@+=feaafiart1ev1aaatCvAUfKttLearuWrP9MDH5MBPbIqV92AaeXatLxBI9gBaebbnrfifHhDYfgasaacH8akY=wiFfYdH8Gipec8Eeeu0xXdbba9frFj0=OqFfea0dXdd9vqai=hGuQ8kuc9pgc9s8qqaq=dirpe0xb9q8qiLsFr0=vr0=vr0dc8meaabaqaciaacaGaaeqabaqabeGadaaakeaafaqabeqadaaabaGaemOCai3aaSbaaSqaaiabdUgaRbqabaGccqGH9aqpdaWcaaqaaiabdogaJnaaBaaaleaacqWGRbWAaeqaaaGcbaGaem4yam2aaSbaaSqaaiabicdaWaqabaaaaaGcbaGaee4DaCNaeeiAaGMaeeyzauMaeeOCaiNaeeyzaugabaGaem4yam2aaSbaaSqaaiabdUgaRbqabaGccqGH9aqpdaWcaaqaaiabigdaXaqaaiabd6eaojabgkHiTiabdUgaRbaadaaeWbqaaiabcIcaOiabdMha5naaBaaaleaacqWG4baEaeqaaOGaeyOeI0IafmyEaKNbaebacqGGPaqkcqGGOaakcqWG5bqEdaWgaaWcbaGaemiEaGNaeyOeI0Iaem4AaSgabeaakiabgkHiTiqbdMha5zaaraGaeiykaKcaleaacqWG4baEcqGH9aqpcqWGRbWAcqGHRaWkcqaIXaqmaeaacqWGobGta0GaeyyeIuoaaaaaaa@5F79@

with *k *= 0, 1, 2..., *N*/4, where *y*_*x *_is the mRNA abundance at the index corresponding to a given *Buchnera *gene location and y¯
 MathType@MTEF@5@5@+=feaafiart1ev1aaatCvAUfKttLearuWrP9MDH5MBPbIqV92AaeXatLxBI9gBaebbnrfifHhDYfgasaacH8akY=wiFfYdH8Gipec8Eeeu0xXdbba9frFj0=OqFfea0dXdd9vqai=hGuQ8kuc9pgc9s8qqaq=dirpe0xb9q8qiLsFr0=vr0=vr0dc8meaabaqaciaacaGaaeqabaqabeGadaaakeaacuWG5bqEgaqeaaaa@2E3F@ is the mean of the *N *gene expression values of *Buchnera*. The standard error of autocorrelation estimates is computed as:

SEk=1N∑i=1k−1ri2
 MathType@MTEF@5@5@+=feaafiart1ev1aaatCvAUfKttLearuWrP9MDH5MBPbIqV92AaeXatLxBI9gBaebbnrfifHhDYfgasaacH8akY=wiFfYdH8Gipec8Eeeu0xXdbba9frFj0=OqFfea0dXdd9vqai=hGuQ8kuc9pgc9s8qqaq=dirpe0xb9q8qiLsFr0=vr0=vr0dc8meaabaqaciaacaGaaeqabaqabeGadaaakeaacqWGtbWucqWGfbqrdaWgaaWcbaGaem4AaSgabeaakiabg2da9maakaaabaWaaSaaaeaacqaIXaqmaeaacqWGobGtaaWaaabCaeaacqWGYbGCdaqhaaWcbaGaemyAaKgabaGaeGOmaidaaaqaaiabdMgaPjabg2da9iabigdaXaqaaiabdUgaRjabgkHiTiabigdaXaqdcqGHris5aaWcbeaaaaa@4074@

Note that the distance between two genes used in this article is the difference of their ranks on the chromosome (approximately equivalent to the number of kb).

In order to analyse the dependence of transcriptional activity as a function of chromosomal position, we also used the spectral density of the autocorrelation function, estimated by Fourier transform techniques. The periodogram obtained by this method illustrates the periodic components, and their corresponding intensities, of the transcriptomic signals along the chromosome of *Buchnera*. The periodic signals of the main periods in *Buchnera *are drawn by averaging and smoothing the signals of all the segments making up each period. Smoothing of the signals was performed by the "Fit Spline" function of the JMP software.

### Statistical analysis

Statistical analyses of microarray data, mean comparisons (ANOVA F-test) and distribution comparisons (Likelihood ratio test) were performed using JMP software. For distribution comparisons, we previously split *Buchnera *gene expression levels into three bins: the 100 most highly expressed genes (labelled as "highly expressed"); the 100 least expressed genes (labelled as "weakly expressed"); and the others (labelled as "moderately expressed").

## Abbreviations

**BAp**: *Buchnera *from the aphid *Acyrthosiphon pisum*

**BBp**: *Buchnera *from the aphid *Baizongia pistaciae*

**BCc**: *Buchnera *from the aphid *Cinara cedri*

**BSg**: *Buchnera *from the aphid *Schizaphis graminum*

**EAAs**: essential amino acids

**gDNA**: genomic DNA

**nBGT**: normalized basal gene transcription

**pTU**: putative transcription units

## Authors' contributions

JV carried out the sample labelling and hybridization, performed the statistical analysis and drafted the manuscript. FC participated in the design of the study, in microarray labelling and hybridization, and in draft manuscript. DR participated in the analysis of periodic transcriptional components. JB carried out the slide preparation and helped with labelling protocols. YR and GF participated in statistical analysis and interpretation of results. JMF participated in the coordination of the study. HC conceived the study, participated in the statistical analysis and help to draft manuscript.

## Supplementary Material

Additional file 1 Analysis of genomic DNA signals hybridized on the *Buchnera *oligo-array. This figure shows the genomic DNA hybridization signals on the *Buchnera *oligo-array. Click here for file

Additional file 2 Distribution of log_2 _mRNA abundances (A) before and (B) after gDNA normalization. This figure compares the distributions of *Buchnera *gene transcription levels before and after genomic DNA normalization. Click here for file

Additional file 3 Organization of genes in the chromosome of *Buchnera aphidicola*. This figure gives information about gene organization in the *Buchnera aphidicola *genome. Click here for file

Additional file 4 Periodograms of *Buchnera *log_2 _normalized mRNA abundances. This figure illustrates periodograms of *Buchnera *normalized mRNA abundances for the original location of genes on the chromosome and according to different simulated permutations of gene positions. Click here for file
