# Supplementary material for: Conservation of the links between gene transcription and chromosomal organization in the highly reduced genome of Buchnera aphidicola
Source: BMC Genomics. 2007 Jun 4;8:143. doi: 10.1186/1471-2164-8-143 (PMC1899503; doi:10.1186/1471-2164-8-143)
Supplement: Additional file 1 — Analysis of genomic DNA signals hybridized on the Buchnera oligo-array. This figure shows the genomic DNA hybridization signals on the Buchnera oligo-array. [file 1471-2164-8-143-S1.pdf]

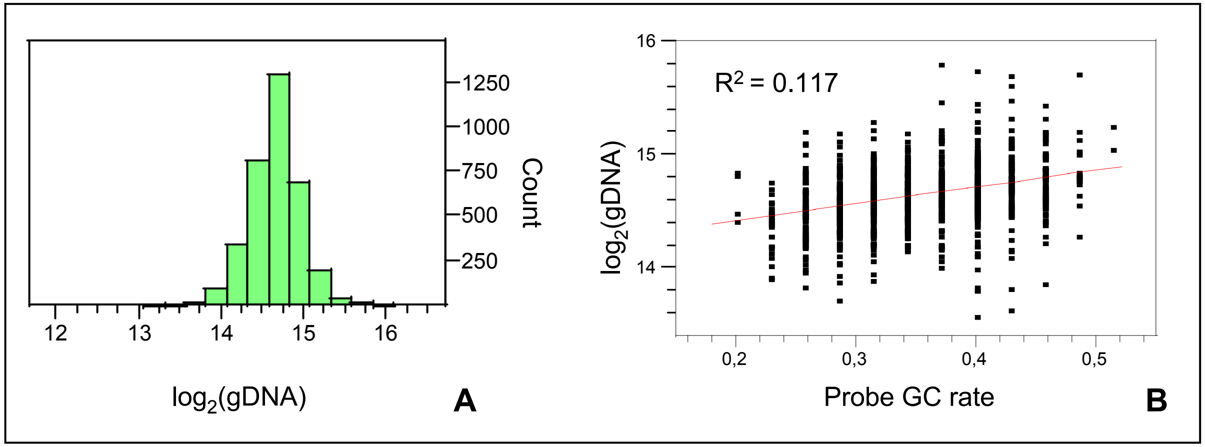

Supplemental Figure 1 - Analysis of genomic DNA signals hybridized on the *Buchnera* oligo-array. (A) Distribution of log_2 fluorescent signals for gDNA hybridization and (B) plot of log_2 gDNA signals versus probe GC rate, Pearson correlation factor (R^2) is specified.
