# Supplementary material for: Conservation of the links between gene transcription and chromosomal organization in the highly reduced genome of Buchnera aphidicola
Source: BMC Genomics. 2007 Jun 4;8:143. doi: 10.1186/1471-2164-8-143 (PMC1899503; doi:10.1186/1471-2164-8-143)
Supplement: Additional file 2 — Distribution of log2 mRNA abundances (A) before and (B) after gDNA normalization. This figure compares the distributions of Buchnera gene transcription levels before and after genomic DNA normalization. [file 1471-2164-8-143-S2.pdf]

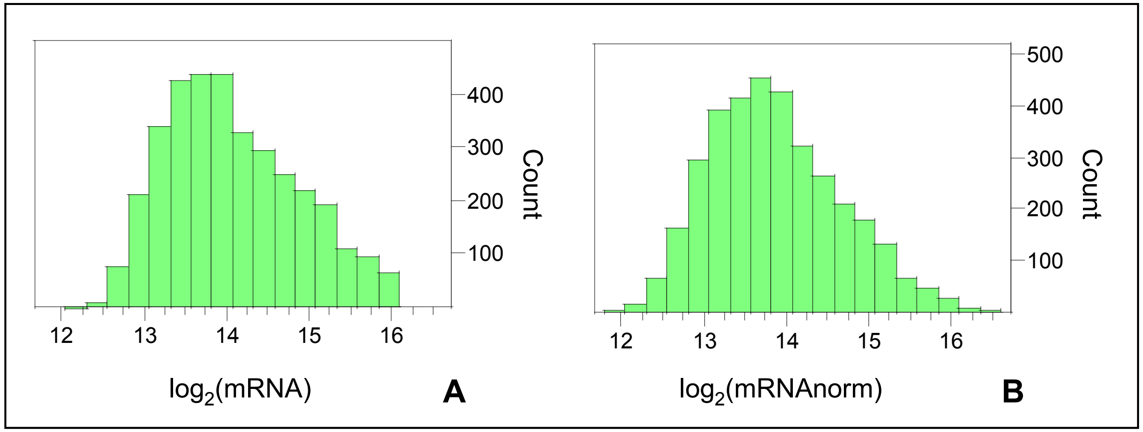

Supplemental Figure 2 - Distribution of $\log_2$ mRNA abundances (A) before and (B) after gDNA normalization.
