# Supplementary material for: Conservation of the links between gene transcription and chromosomal organization in the highly reduced genome of Buchnera aphidicola
Source: BMC Genomics. 2007 Jun 4;8:143. doi: 10.1186/1471-2164-8-143 (PMC1899503; doi:10.1186/1471-2164-8-143)
Supplement: Additional file 3 — Organization of genes in the chromosome of Buchnera aphidicola. This figure gives information about gene organization in the Buchnera aphidicola genome. [file 1471-2164-8-143-S3.pdf]

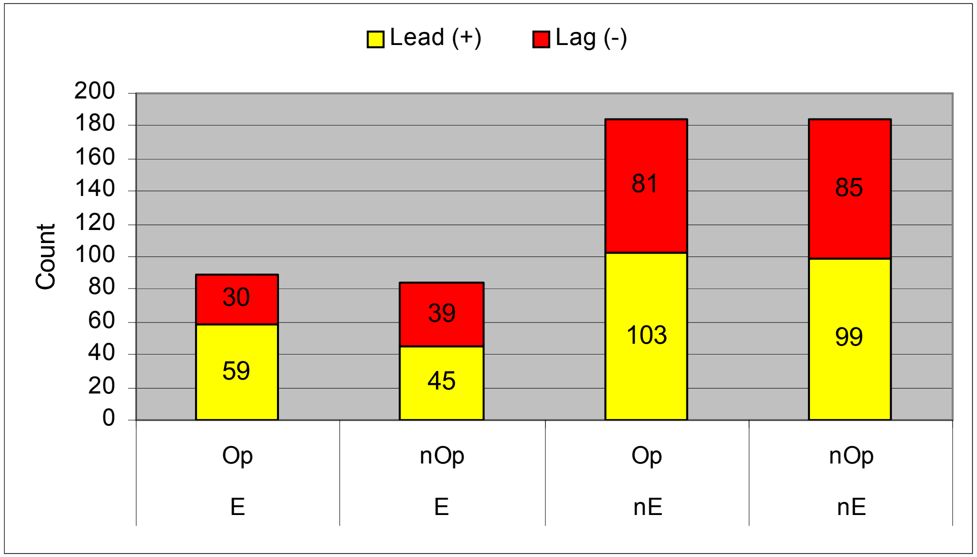

Supplemental Figure 3 - Organization of genes in the chromosome of *Buchnera aphidicola*. This figure shows the distribution of the genes between the leading ("+") and the lagging strand DNA ("-") for the essential genes ("E") and the non-essential ones ("nE"), predicted to be in operons ("Op"), or not ("nOp").
