# Supplementary material for: Conservation of the links between gene transcription and chromosomal organization in the highly reduced genome of Buchnera aphidicola
Source: BMC Genomics. 2007 Jun 4;8:143. doi: 10.1186/1471-2164-8-143 (PMC1899503; doi:10.1186/1471-2164-8-143)
Supplement: Additional file 4 — Periodograms of Buchnera log2 normalized mRNA abundances. This figure illustrates periodograms of Buchnera normalized mRNA abundances for the original location of genes on the chromosome and according to different simulated permutations of gene positions. [file 1471-2164-8-143-S4.pdf]

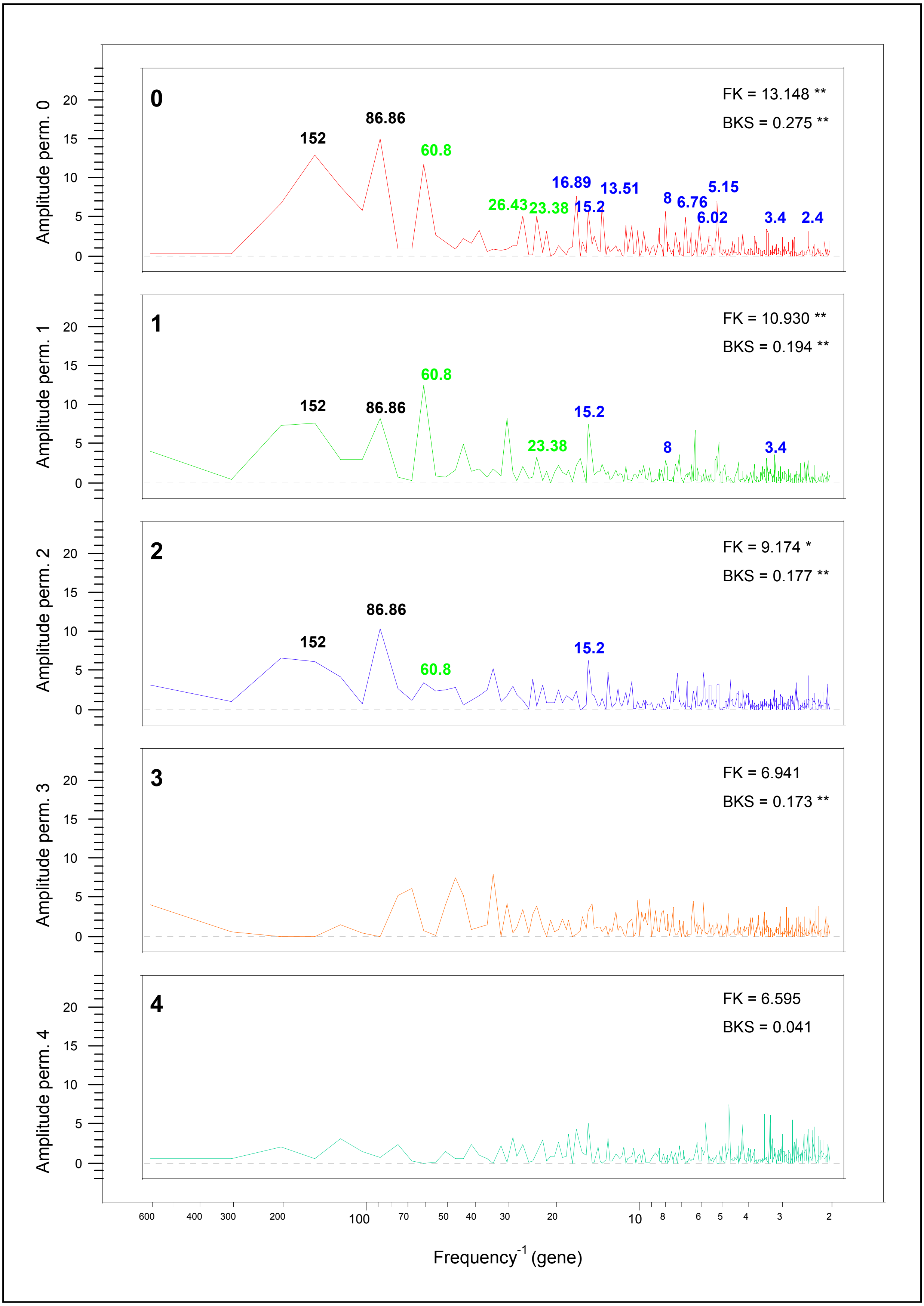

Supplemental Figure 4 - Periodograms of *Buchnera* $\log_2$ normalized mRNA abundances. (0) shows the periodogram for the original location of genes on the chromosome, and (1) to (4) show the periodograms corresponding to the different simulated permutations of gene positions presented in Figure 6. The main periods of the original periodogram are specified. Results for Fisher's Kappa test (FK) and Bartlett's Kolmogorov-Smirnov test (BKS) are marked with "**" (P-val < 0.01) or with "*" (P-val < 0.05), when they are significant.
